# The Effect of Virtual Reality-Based Therapy on Improving Upper Limb Functions in Individuals With Stroke: A Randomized Control Trial

**DOI:** 10.3389/fnagi.2021.731343

**Published:** 2021-11-02

**Authors:** Ehab Mohamed Abd El-Kafy, Mansour Abdullah Alshehri, Amir Abdel-Raouf El-Fiky, Mohamad Abdelhamid Guermazi

**Affiliations:** ^1^Department of Physiotherapy, Faculty of Applied Medical Sciences, Umm Al-Qura University, Mecca, Saudi Arabia; ^2^School of Health and Rehabilitation Sciences, The University of Queensland, Brisbane, QLD, Australia; ^3^Department of Rheumatology, King Abdul-Aziz Hospital, Jeddah, Saudi Arabia

**Keywords:** virtual reality, exergames, physiotherapy, upper limb (UL), stroke

## Abstract

**Background:** Stroke is a common cause of motor disability. The recovery of upper limb after stroke is poor, with few stroke survivors regaining some functional use of the affected upper limb. This is further complicated by the fact that the prolonged rehabilitation is accompanied by multiple challenges in using and identifying meaningful and motivated treatment tasks that may be adapted and graded to facilitate the rehabilitation program. Virtual reality-based therapy is one of the most innovative approaches in rehabilitation technology and virtual reality systems can provide enhanced feedback to promote motor learning in individuals with neurological or musculoskeletal diseases.

**Purpose:** This study investigated the effect of virtual reality-based therapy on improving upper limb functions in individuals with chronic stroke.

**Methods:** Forty Saudi individuals with chronic stroke (6–24 months following stroke incidence) and degree of spasticity ranged between 1, 1 + and 2 according to Modified Ashworth Scale were included in this study. Participants were randomly assigned into two groups, experimental and control, with the experimental group undertaking a conventional 1-h functional training program, followed by another hour of virtual reality-based therapy using Armeo Spring equipment and the control group received 2 h of a conventional functional training program. The treatment program was conducted three times per week for three successive months. The change in the scores of Action Research Arm Test (ARAT), Wolf Motor Function Test (WMFT), WMFT-Time (time required to complete the test) and Hand Grip Strength (HGS) were recorded at baseline and after completion of the treatment. Parametric (paired and unpaired *t*-tests) non-parametric (Wilcoxon and Mann–Whitney tests) statistical tests were used to identify the differences within and between groups (experimental group and control group) and evaluation times (pre- and immediately post-treatment).

**Results:** Both groups showed significant differences (all, *P* < 0.05) in all measured variables after 3 months of the treatment. Individuals with stoke in the experimental group had a better improvement in ARAT (*P* < 0.01), WMFT (*P* < 0.01) and WMFT-Time (*P* < 0.01) scores after completion of the treatment compared to the control group. No significant difference in HGS scores was detected between groups after completion of the treatment (*P* = 0.252).

**Conclusion:** The use of combined treatment of virtual reality-based therapy and conventional functional training program is more effective for improving upper limb functions in individuals with chronic stroke than the use of the conventional program alone.

## Introduction

Stroke is an acute event that primarily involves neurological damage leading to disability and mortality (Sabut et al., 2010; Sacco et al., 2013). Globally, it is the 2nd or 3rd most frequent cause of death as well as one of the main causes of acquired adult disability (Langhorne et al., 2011). In Saudi Arabia (SA), the incidence of stroke in adults is approximately 30–40 per 100,000 population annually (Qureshi, 2008; Alahmari and Paul, 2016), with an estimated 20,000 new strokes, 8,000 disabilities and 4,000 deaths (Al Khathaami et al., 2011). Multiple impairments are observed including weakness, fatigue, alterations in tone, sensory loss, cardiovascular deconditioning, uncoordinated responses, poor balance and difficulty in walking, which might impact on the ability to perform functional activities in those who experience a stoke (Guy et al., 2004; Langhorne et al., 2009).

Changes in the affected upper limb are often more pronounced than those in the affected lower limb (Wiklund and Uvebrant, 1991) and include functional limitations of the affected arm and slow uncoordinated motion of the hand (Brown and Walsh, 2000; Chin et al., 2020). There are also common changes such as the difficulty performing reaching tasks due to changes in the timing and coordination with abnormal postural adjustments (Hung et al., 2004; Steenbergen and Van Der Kamp, 2004) or inability to control grasping, finger-tip force and timing during manipulation of an object (Duff and Gordon, 2003; Gordon et al., 2003). Stroke patients often experience difficulties in participation in home, work, community life (Sköld et al., 2004) or difficulty performing daily living activities (ADL) such as feeding, dressing, and grooming due to a combination of physical, cognitive and perceptual problems (Mayo et al., 1999; Sköld et al., 2004).

The functional performance of the affected upper limb can be improved when stroke patients have sufficient opportunities to practice (Walker et al., 2000). There are different techniques/approaches that can be used in the management such as physiotherapy, occupational therapy, conductive education, splinting, casting pharmacotherapy and surgery or specific techniques such as neurodevelopmental therapy or constrained induced movement therapy (Sakzewski et al., 2009). However, there is no strong evidence of successful treatment with any of these techniques/approaches.

Virtual reality is the usage of interactive simulations created by computers to provide useful experiences for individuals by engaging and interacting within three-dimensional environments that are similar to objects and events of the real world (Sisto et al., 2002). The use of virtual reality for rehabilitation of stroke patients has been shown to be interactive and enjoyable, and may help to improve the motor control and function of upper limb with sufficient use (Holden et al., 2007; Kim et al., 2018). Using virtual reality in rehabilitation (known as virtual reality-based therapy) is one of the most innovative developments in rehabilitation technology, as virtual environments are a promising future approach in the rehabilitation and improvement of ADL after stroke. Virtual reality-based therapy has the potential to be used in a range of settings including patients’ homes or nursing homes that allows additional practice outside formal rehabilitation sessions (Crosbie et al., 2007; Kim et al., 2020).

Few studies are available that have used virtual reality for rehabilitation in individuals with stroke and no study has investigated the use and effects of virtual reality-based therapy in Saudi individuals with stroke. Accordingly, further studies are required to prove the efficiency of virtual reality based training for improvement of the upper limb functions in patients with stroke in Saudi Arabia. Therefore, this study aimed to investigate the effect of virtual reality-based therapy on improving the functions of upper limb in comparison with the conventional functional training program in Saudi chronic stroke patients.

Few studies are available that have used virtual reality for rehabilitation in individuals with stroke and no study has investigated the use and effects of virtual reality-based therapy in Saudi individuals with stroke. In SA, stoke incidence is expected to increase and is becoming a critical problem and an important cause of mortality and morbidity and also impact on performing everyday activities in Saudi population. Therefore, treatments that can provide a quick and better improvement for upper limb functions in Saudi patients with stroke are necessary. Therefore, this study aimed to investigate the effect of virtual reality-based therapy on improving the functions of upper limb in comparison with the conventional functional training program in Saudi chronic stroke patients.

## Materials and Methods

### Participants

A total of 62 chronic stroke patients were screened for inclusion in this study, with only 40 participants meeting the inclusion criteria. Table 1 shows the characteristics of the included participants.

**TABLE 1:** Participants’ characteristics, number of affected dominant limb and spasticity degree.

	Control group (*n* = 20)	Experimental group (*n* = 20)
**Participants’ characteristics**		
Age (years)	53.32 ± 5.13	54.46 ± 4.27
Sex (number; Male/Female)	15/5	16/4
Height (cm)	167.75 ± 5.69	168.62 ± 5.27
Weight (kg)	86.86 ± 4.47	87.41 ± 4.26
**Dominant upper limb**		
Number of affected dominant upper limb	17	16
**Spasticity degree (Modified Ashworth Scale)**		
1	5	2
1+	11	13
2	4	5

The inclusion criteria were adult participants aged 50–60 years with a confirmed diagnosis of chronic stroke (at least 6 months following the stroke incidence) secondary to ischemia or hemorrhage. The degree of spasticity of the affected upper limb ranged between 1, 1 + and 2 according to Modified Ashworth Scale (Rw and Smith, 1987; Sloan et al., 1992). All participants had the ability to extend the wrist at least 20° and the fingers for 10° from the full flexion, allowing participants to engage in performing functional activities. Also, they were cognitively able to understand and follow instructions and did not receive other treatments to improve the functions of affected upper limb except the treatment provided in this study.

Any participant with a cognitive reduction (<23 points based on Mini-Mental State Examination scale) was excluded (Pangman et al., 2000). Participants with fixed contractures and stiffness in upper limb joints (e.g., shoulder, elbow, wrist, and fingers joints), painful shoulder syndrome, and participants who had major rotational malalignments in the affected upper limb were also excluded from this study. Other exclusion criteria were participants with a cardiac pacemaker, visual, auditory, and perceptual diseases/impartments, uncontrolled seizures, those who received botulinum toxin (6 months before the beginning of the study) or muscle-tone control medication (3 months before the beginning of the study).

This study was approved by the Biomedical Ethics Committee, Umm Al-Qura University, Mecca, Saudi Arabia and conducted in the Physiotherapy Department of Umm Al-Qura University. The participants were recruited from the western region (Mecca and Jeddah cities) and provided written informed consent authorizing their participation. This study was registered in ClinicalTrials.gov (NCT04764994).

### Study Design and Randomization

**FIGURE 1:**
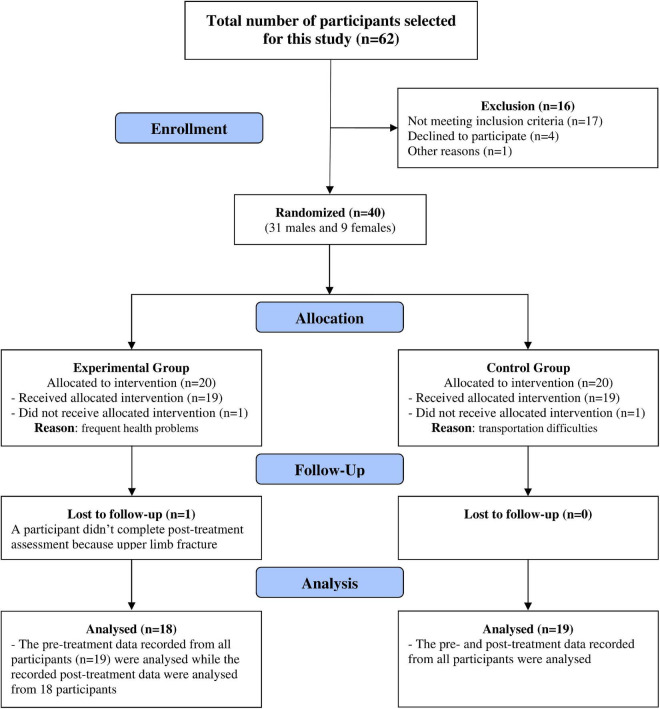
Flow diagram of the study.

The study design was a two-armed randomized control trial. The randomization of the participants in both treatment groups (experimental and control) was performed using a computerized sequence generation. Figure 1 shows the recruitment process and the flow of participants throughout the study.

Given the nature of the treatment in this study, it was not possible for therapists and participants to be blinded but both therapists and participants were blinded to the randomization process to ensure the maximum degree of credibility of the obtained results. The assessment of the stroke patients was performed by assessors who had not participated in the application of treatment and were blinded to the randomization process. The participants were allocated in two groups of twenty patients, experimental and control groups.

### Assessment

Outcome measures were based on the following tests: (1) Action Research Arm Test, (2) Wolf Motor Function Test (WMFT), and (3) Hand Grip Strength (HGS). The change in the scores of these tests at baseline and after completion of the treatment were recorded. The assessment was performed by blinded assessors who did not engage in the treatment program and did not know which group each participant was in. The used outcome measures of this study can be linked to the “neuromusculoskeletal and movement-related functions” domain of “Body Functions and Structures” component of ICF classification (The International Classification of Functioning, Disability and Health).

Action Research Arm Test was used to assess the changes in limb functions including ability to handle objects with different size, weight, and shape. It is highly recommended as a clinical and research tool for assessing the changes in motor impairments of the upper limbs following stroke (Song, 2012; Carpinella et al., 2014; Nomikos et al., 2018; Dandekar and Ganvir, 2019; Spence et al., 2020). This test can be considered as an arm-specific measure of activity limitation and consists of nineteen items in four subscales: grasp, grip, pinch, and gross movements. Each item was scored on a four-level ordinal scale 0–3: cannot perform any part of the test (score = 0), performs partially (score = 1), takes a long time to complete the test (score = 2) and performs the test normally (score = 3). The total score ranged from 0 to 57, with a low score indicating no movements can be performed while the highest score indicates normal functional performance of upper limb (Lyle, 1981).

Wolf Motor Function Test is a tool with acceptable test-retest reliability to assess the motor ability of the upper limb in participants with stroke through timed and functional tasks (Morris et al., 2001; Nijland et al., 2010; Hodics et al., 2012). It consists of fifteen items, six items related to timed joint-segment movements tasks and nine items related to integrative functional movements tasks. The assessors tested the less affected upper limb first, followed by the most affected side. The fifteen timed items were rated on a 6-point scale (0–5): the participant is unable to use upper limb (score = 0), or the participant can use upper limb and its movement appears to be normal (score = 5). A maximum of 120 s was allocated to each item. The total score ranged from 0 to 75, with a lower score indicates lower functioning levels (Wolf et al., 2001). In addition, the time required to complete the test in seconds (WMFT-Time) was measured.

Hand Grip Dynamometer (BTE Technologies, Hanover, MD, United States) was used to assess the change in the strength of the hand muscles of the affected upper limb (in pounds). The higher the score of the hand grip after completion of the treatment program compared to the baseline score, the better the improvement of hand functional abilities.

### Treatment Protocol

The treatment was delivered face-to-face and provided individually in three sessions per week for 3 months for both experimental and control groups. Every treatment session lasted 2 h with a 15-min rest between the first and second hours.

Participants in the experimental group underwent a three-part treatment program. The first part involved muscle facilitation exercises, proprioceptive neuromuscular facilitation exercises, strengthening activities, stretching exercises and postural reactions exercises. The second part included arm-reaching tasks, arm-hand tasks, manipulative tasks (grasping and release activities) with the inclusion of the more affected upper limb in functional tasks of ADL. The first and second parts were applied for 1 h, followed by 15 min rest, then the third part was applied for 1 h. The third part included a virtual reality-based training program using Armeo Spring to simulate a range of upper limb tasks related to arm-reaching to target, reach and grasp (arm-hand activities) and manipulative tasks through using different games as appropriate. Armeo Spring (MRF; Hocoma, Switzerland) is a functional device used for upper limb rehabilitation and can provide a specific therapy with augmented feedback. It facilitates intensive task-oriented upper limb therapy in neurological diseases and injuries such as stroke and traumatic brain injury. This device involves an adjustable arm support with augmented feedback and a large 3D workspace allowing to perform functional therapy exercises in a virtual reality environment.

The control group underwent a conventional functional training program (conventional physiotherapy program) for 2 h comprising two parts those provided to the experimental group but each part lasted for 1 h in the control group with 15 min rest in between.

The provided conventional functional training program in both groups focused on enhancement of the following functions and skills of the affected upper limb: (1) shoulder abduction and external rotation, (2) elbow extension, (3) forearm supination, (4) wrist radial deviation, and (5) thumb and fingers extension and abductions.

### Power Analysis

To identify the required sample size, a preliminary power analysis was calculated. The power was set at 80%, alpha (α) was at 0.05 and the effect size was set at 0.5; considering two treatment groups and two different evaluation times. Subsequently, the power analysis revealed that the required sample size of this study was 40 participants.

### Statistical Analysis

For parametric data, paired *t*-tests (paired samples) were used to compare the changes in WMFT-Time and HGS between pre- and immediately post-treatment within each group. Unpaired *t*-tests (independent samples) were used to compare between groups (experimental and control groups) at baseline and immediately after completion of the treatment. The results were expressed as mean ± standard deviation (SD). For non-parametric data, Wilcoxon tests were used to compare the changes in ARAT and WMFT between pre- and immediately post-treatment within each group. The Mann–Whitney test was used to compare between groups at baseline and immediately after completion of the treatment. The results were expressed as mean rank. SPSS (version 26) was used to analyze the data and *P* values less than 0.05 were considered significant.

## Results

There were significant differences (all, *P* < 0.01) in the mean scores of ARAT, WMFT, and WMFT-Time between pre- and post-treatment for each group (experimental and control groups) but no significant differences were detected between groups at baseline (Table 2). After completion of the treatment, significant differences were detected in favor of the experimental group in these tests (all, *P* < 0.01).

**TABLE 2:** Within- and between- group differences for upper-limb functional measures at baseline and immediately after 12 weeks of treatment.

Parameter	Control group	Experimental group	*P-*value
**Action Research Arm Test**			
Mean Rank–Pre	17.72	18.29	0.868
Mean Rank–Post	12.49	23.35	0.0034
*P*-value	0.0027	0.0011	–
**Wolf Motor Function Test**			
Mean Rank–Pre	18.47	17.50	0.778
Mean Rank–Post	11.83	24.35	0.0015
*P*-value	0.0031	0.0014	–
**Wolf Motor Function Test–Time (in seconds)**			
Mean ± SD–Pre	47.50 ± 4.77	47.94 ± 4.86	0.742
Mean ± SD–Post	41.39 ± 3.80	36.71 ± 4.19	0.0014
*P*-value	0.0017	0.0001	–
**Hand Grip Strength (in pounds)**			
Mean ± SD–Pre	10.72 ± 1.93	10.59 ± 2.06	0.844
Mean ± SD–Post	11.94 ± 2.18	12.88 ± 2.57	0.252
*P*-value	0.0183	0.0241	–

*SD, standard deviation.*

For HGS, there was a significant difference between pre- and post-treatment for each group (all, *P* < 0.05) but no differences were detected between groups at baseline and after the completion of treatment (Table 2).

## Discussion

The present study aimed to investigate whether virtual reality-based therapy had a beneficial effect on improving the functions of upper limb in chronic stroke patients and compare its effect with that of the conventional physiotherapy program. The results revealed that virtual reality-based therapy combined (with conventional physiotherapy program) greatly improved upper limb functions in chronic stroke patients compared to the use of a conventional physiotherapy program alone.

The poor recovery of the affected upper limb after stroke can be explained by the phenomenon of learned non-use which can be easily developed after stroke and may lead to the development of secondary complications such as weakness or atrophy of the muscles, imbalance or tightness of the muscles, stiffness or deformity of upper limb joints, poor circulation to the upper limb and pain (Botte et al., 1988; Feys et al., 1998). These factors may cause reluctance to use the affected arm that might impede the recovery of motor functions. Therefore, any physical therapy programs to promote the utilization of the affected upper limb will certainly overcome this phenomena, allowing patients to use their upper limbs and accelerate functional recovery in stroke patients.

Therefore, the application of the conventional physiotherapy program in this study may explain the significant improvement that occurred in both groups after treatment. This training program encouraged the participants, providing them with the opportunity to use their affected upper limbs in different purposeful and meaningful activities, thereby augmenting the process of re-learning through facilitation of sensory-motor experience, controlling muscle contraction, coordination of neuromuscular activities, as well as improving the range of motion of joints in the affected upper limb.

In contrast to conventional face-to-face therapy, stroke survivors can be trained to practice virtual tasks independently which can increase the amount of time the patients spend in activities that may lead to better ADL outcomes (Kwakkel et al., 2004). The better results that were gained in the experimental group than in the control group might be attributed to the application of virtual reality-based therapy that allowed the participants in this group to perform motor activities in more enjoyable environments, manipulate items/objects virtually which motivated them to continue therapy.

The positive impact of the virtual reality-based therapy on improving motor functions has been supported in multiple previous studies as it can provide participants the capacity to practice movements independently in more challenging tasks and more motivating environments, stimulate patients to concentrate and exercise more control over the environment compared with real-life settings, as well as enable therapists to grade the task to the appropriate level of challenge and increase the intensity of training while providing augmented three-dimensional and direct sensorial feedback (Dobkin, 2004; Piron et al., 2005; Merians et al., 2006; Mihelj et al., 2012). It can also provide mechanical-assistance to the trained upper limb which partially relieves its weight enabling patients to use repetitive and active exertion of goal-directed movements with larger ROM and greater multi-joint coordination during the practice of complex motor tasks in an enriched learning environment (Beer et al., 2007; Timmermans et al., 2009). Furthermore, it can improve the ability of patients to address spatial and temporal accuracy necessary for the movement to meet environmental demands (Ada and Canning, 2005; Taveggia et al., 2016).

The significant effect of virtual reality-based therapy on improving motor performance in individuals with chronic stroke in the present study was in line with the findings of Merians et al. (2002); Kwakkel et al. (2008), Basteris et al. (2014), and Levac et al. (2019) who demonstrated that repetitive movements in real environments using robot-assisted arm rehabilitation and virtual reality therapy were effective in improving upper limb functions, positively enhancing the performance of ADL and better than those who trained with conventional treatment methods (Merians et al., 2002; Kwakkel et al., 2008; Basteris et al., 2014; Levac et al., 2019).

There are some limitations in this study that should be acknowledged. The first limitation is the difficulty to recruit more participants due to COVID-19 pandemic as some stoke patients that met inclusion criteria refused to participate in this study. The second limitation is due to not fully implementing the ICF classification. The third limitation is due to the demands placed on participants and their age as researchers did not apply measurements to examine other functional activities such as walking. The fourth limitation is that the long-term effect of virtual reality-based training for improving the functions of affected upper limb in stroke had not yet been established.

Future studies to consider different stroke stages (acute, subacute or chronic) and age groups, and to identify the ideal duration of treatment session and the optimal program length to obtain the maximum benefits from virtual reality-based treatment are recommended.

## Conclusion

Combined treatment of virtual reality-based therapy and conventional physiotherapy program is more effective for improving upper limb functions in chronic stroke patients than the use of conventional physiotherapy program alone.

## Data Availability Statement

The original contributions presented in the study are included in the article/supplementary material, further inquiries can be directed to the corresponding author/s.

## Ethics Statement

The studies involving human participants were reviewed and approved by this study was approved by the Biomedical Ethics Committee (HAPO-02-K-012-2020-10-473), Umm Al-Qura University, Mecca, Saudi Arabia and conducted in the Physiotherapy Department of Umm Al-Qura University. The patients/participants provided their written informed consent to participate in this study.

## Author Contributions

EE-K developed the study protocol and designed the study methods. EE-K, AE-F, and MG collected the data of the study. EE-K and AE-F performed the data and statistical analysis. EE-K, MA, AE-F, and MG cooperatively did the data presentation and interpretation. EE-K and MA drafted the manuscript. All authors read, revised, and approved the final version of the manuscript.

## Conflict of Interest

The authors declare that the research was conducted in the absence of any commercial or financial relationships that could be construed as a potential conflict of interest. The reviewer FH declared a past co-authorship with several of the authors, MA and AE-F, to the handling editor.

## Publisher’s Note

All claims expressed in this article are solely those of the authors and do not necessarily represent those of their affiliated organizations, or those of the publisher, the editors and the reviewers. Any product that may be evaluated in this article, or claim that may be made by its manufacturer, is not guaranteed or endorsed by the publisher.
